# A Three-Terminal Si-Ge Avalanche Photodiode with a Breakdown Voltage of 6.8 V and a Gain Bandwidth Product of 1377 GHz

**DOI:** 10.3390/mi16111222

**Published:** 2025-10-27

**Authors:** Chao Cheng, Jintao Xue, Xishan Yu, Jifang Mu, Binhao Wang

**Affiliations:** 1 State Key Laboratory of Ultrafast Optical Science and Technology, Xi’an Institute of Optics and Precision Mechanics, Chinese Academy of Sciences, Xi’an 710119, China; chengchao2022@opt.ac.cn (C.C.); xuejintao@opt.ac.cn (J.X.); yuxishan24@mails.ucas.ac.cn (X.Y.); wangbinhao@opt.ac.cn (B.W.); 2 Photoelectric Tracking and Measurement Technology Laboratory, Xi’an Institute of Optics and Precision Mechanics, Chinese Academy of Sciences, Xi’an 710119, China; 3 School of Future Technology, University of Chinese Academy of Sciences, Beijing 100049, China

**Keywords:** avalanche photodiode, silicon nitride, silicon-on-insulator

## Abstract

Silicon–germanium (Si-Ge) avalanche photodiodes (APDs), fully compatible with complementary metal–oxide–semiconductor (CMOS) processes, are critical devices for high-speed optical communication. In this work, we propose a three-terminal Si-Ge APD on a silicon-on-insulator (SOI) substrate based on device simulation studies. The proposed APD employs a separate absorption and multiplication structure, achieving an ultra-low breakdown voltage of 6.8 V. The device operates in the O-band, with optical signals laterally coupled into the Ge absorption layer via a silicon nitride (Si_3_N_4_) waveguide. At a bias of 2 V, the APD exhibits a responsivity of 0.85 A/W; under a bias of 6.6 V, it achieves a 3-dB optoelectronic (OE) bandwidth of 51 GHz, a direct current gain of 27, and a maximum gain–bandwidth product (GBP) of 1377 GHz. High-speed performance is further confirmed through eye-diagram simulations at 100 Gbps non-return-to-zero (NRZ) and 200 Gbps four-level pulse amplitude modulation (PAM4). These results clearly show the strong potential of the proposed APD for optical communication and interconnect applications under stringent power and supply voltage constraints.

## 1. Introduction

With the rapid advancement of artificial intelligence (AI), machine learning, and cloud computing, the demand for computing power and high-speed data transmission in large-scale data centers and high-performance computing (HPC) systems has grown exponentially [1]. However, traditional electrical interconnects suffer from high latency and excessive power consumption in long-distance transmission, making them increasingly inadequate for modern applications. In contrast, optical interconnects offer significant advantages, with latency and power consumption largely independent of transmission distance [2,3]. To address these challenges, various photonic integration platforms have been developed across different material systems, including group-III–V compounds such as indium phosphide (InP) [4] and gallium arsenide (GaAs) [5], group-IV silicon photonics (SiPh) [6,7,8] and planar lightwave circuits (PLCs) [9], as well as lithium niobate (LiNbO_3_) [10]. Among them, silicon photonics (SiPh), fully compatible with standard CMOS processes, enables low-cost and high-density integration and is widely recognized as a key enabler for next-generation communication systems and data transmission infrastructure [11].

As a key component in optical receiver systems, the optical receiver typically comprises fundamental modules such as couplers, demultiplexers, and APDs [12]. The performance of the APD directly determines the overall sensitivity and signal quality of the receiver, making the development of high-performance APDs fully compatible with the SiPh platform a critical research focus. Compared with Si-based demultiplexers, Si_3_N_4_ waveguides exhibit excellent polarization-insensitive characteristics due to their highly symmetric structure and moderate refractive index contrast with the silicon dioxide (SiO_2_) cladding. In recent years, three-dimensional integrated multilayer Si_3_N_4_ waveguides based on the SiPh platform have been extensively investigated and experimentally demonstrated [13,14,15]. Edge coupler fabricated on Si_3_N_4_ platform exhibits excellent performance, with insertion losses below 0.5 dB per facet. However, when such demultiplexers are integrated with APDs coupled to Si waveguides, an additional transition structure is typically required to transfer the optical signal from the Si_3_N_4_ waveguide to the Si waveguide before detection [16]. This extra coupling step inevitably introduces additional insertion loss and polarization dependence, thereby limiting the overall system performance and integration density.

APDs with separate absorption and multiplication regions can take advantage of the high absorption efficiency of Ge in the O- and C-bands and the low impact ionization coefficient ratio of Si [17,18,19]. However, such devices typically require relatively high operating voltages, and most reported Si-Ge APDs operate above 10 V [20,21,22,23]. Considering that the maximum supply voltage in modern computer architectures is 12 V and that a safety margin is required in practical applications, a high APD breakdown voltage limits its applicability and increases the risk of breakdown; therefore, the breakdown voltage is preferably kept below 10 V. Fortunately, with continued in-depth research, it has been found that Si-Ge APDs with a narrow multiplication region exhibit a low temperature sensitivity of breakdown voltage. This characteristic offers a promising prospect for developing thermally robust and reliable APD devices for optical interconnects in future data centers [24,25]. Motivated by this potential, increasing attention has been devoted to reducing the breakdown voltage of such devices. To address this issue, previous studies have directly incorporated the avalanche multiplication process into the Ge region, successfully reducing the operating voltage below 10 V. Although this approach significantly lowers the bias, it often leads to increased noise, and its practical feasibility is still under investigation. More recently, three-terminal electrode structures have been proposed to further reduce the operating voltage, enabling APD operation at around 6 V and offering a more feasible solution. However, the device employs an interdigitated “finger” structure with N- and P-type doping. This doping configuration increases the PN junction area, adversely affecting the device’s electrical parasitics and limiting its bandwidth, thereby constraining its potential for high-speed optical communication applications [26].

In this work, we present, for the first time, a three-terminal Si-Ge APD with lateral coupling via a Si_3_N_4_ waveguide. The optical signal is laterally coupled into the Ge absorption region from both sides through the Si_3_N_4_ waveguide, resulting in a more uniform optical field distribution within the Ge layer. Furthermore, the Si_3_N_4_-based lateral coupling scheme enables efficient integration with Si_3_N_4_-based wavelength-division multiplexers (WDMs). The choice of Si_3_N_4_ platform for the demultiplexer is motivated by its superior capability to achieve polarization-insensitive operation and lower insertion loss compared with demultiplexers fabricated on conventional silicon platforms [27]. In the electric field design, we introduce an innovative P+ type doping scheme in the Si waveguide beneath the Ge absorption layer, effectively shortening the carrier transit path and significantly enhancing the device’s 3-dB OE bandwidth. By independently controlling the electric fields in the Ge absorption region and the multiplication region, the avalanche breakdown voltage of the device is significantly reduced to 6.8 V. As a result of these design optimizations, the APD achieves a 3-dB OE bandwidth of 51 GHz and a multiplication gain of 27 under a reverse bias voltage of 6.6 V, while exhibiting a responsivity of 0.85 A/W at 2 V. To further validate the high-speed transmission performance, eye diagram simulations were conducted at 100 Gbps using NRZ modulation and at 200 Gbps using PAM4, both of which show clearly open eyes. These results indicate that the proposed APD holds considerable potential for low-bias voltage optical communication system applications.

## 2. APD Design

**Figure 1:**
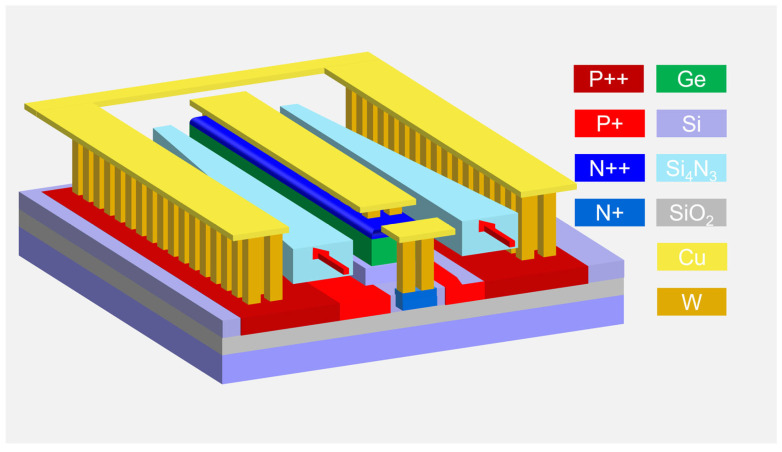
Three-dimensional schematic of Si-Ge APD.

Figure 1 illustrates the three-dimensional (3D) schematic of the proposed APD. A lateral coupling scheme via a Si_3_N_4_ waveguide is employed to efficiently couple the evanescent field into the Ge absorption region. The incident direction of the optical field is indicated by the red arrow. Compared to Si waveguides, Si_3_N_4_ waveguides offer four notable advantages [28,29]: (1) they can tolerate higher input optical power, thereby reducing the risk of waveguide damage under high-power input; (2) they exhibit lower propagation loss and smaller polarization-dependent loss, which helps mitigate the polarization sensitivity of the device; (3) they readily enable lateral coupling, allowing light to be coupled simultaneously from both sides into the Ge absorption region, thereby achieving a more uniform optical field distribution; and (4) this design enables efficient integration with polarization-insensitive Si_3_N_4_-based WDMs without requiring additional converters, such as vias from Si to Si_3_N_4_ waveguides.

**Figure 2:**
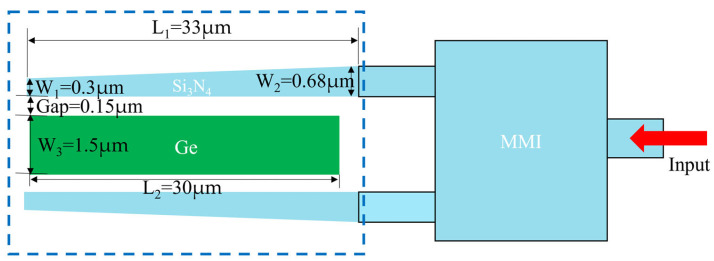
Simplified structural diagram of Si-Ge APD.

The operating principle of the APD is illustrated in Figure 2. The optical signal is guided through the Si_3_N_4_ waveguide to a conventional 1 × 2 Si_3_N_4_ multi-mode interference (MMI), where it is split and subsequently absorbed by the Ge layer to generate photocarriers. Optical simulations were performed using three-dimensional finite-difference time-domain (3D-FDTD) methods, and the device parameters were designed with reference to the standard 130 nm SOI SiPh platform. After optimizing device performance, the determined optical structure parameters are W_1_ = 0.3 µm, W_2_ = 0.68 µm, L_1_ = 33 µm, Gap = 0.15 µm, L_2_ = 30 µm, and W_3_ = 1.5 µm.

**Figure 3:**
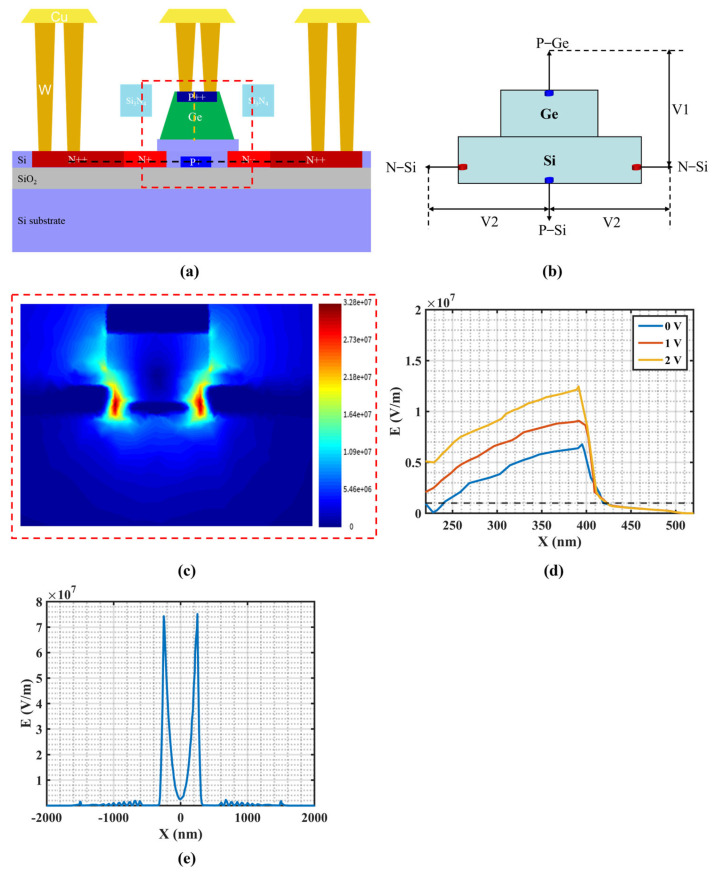
(**a**) Cross-sectional doping profile of the Si-Ge APD. (**b**) The simplified schematic of the three-terminal APD. (**c**) The electric field distribution in the region enclosed by the red dashed box. (**d**) Electric field along the orange dashed line in the P-Ge region under different reverse bias voltages. (**e**) Electric field along the black dashed line in the Si region at a bias voltage of 6.6 V.

After completing the optical field design of the APD, the doping profile was further optimized. The overall 3D device structure is shown in Figure 1. A P^+^ doping layer is introduced in the Si waveguide beneath the Ge absorption layer, with an additional third electrode connected to the P^+^ region to control the avalanche electric field. The cross-sectional view of the device doping profile is illustrated in Figure 3a, where heavy doping is applied at metal-contact regions to reduce resistance. The doping concentrations are N++ (1.0 × 10^20^ cm^−3^), N+ (1.0 × 10^19^ cm^−3^), P++ (7.1 × 10^19^ cm^−3^), and P+ (5.3 × 10^18^ cm^−3^), respectively.

Figure 3b illustrates the simplified schematic of the three-terminal APD, where the three terminals—P-Ge, N-Si, and P-Si—enable independent control of two internal voltages. The voltage V1 applied between P-Ge and N-Si controls the electric field strength in the Ge region, ensuring that photogenerated carriers drift at their saturation velocity while preventing avalanche multiplication in Ge. The voltage V2 applied between N-Si and P-Si regulates the electric field strength in the Si region, thereby confining the avalanche region within the Si waveguide. Figure 3c shows the electric field distribution within the red dashed box at a 6.6 V reverse bias, with the avalanche region confined in the Si waveguide. Figure 3d shows the electric field along the orange dashed line in the P-Ge region at different reverse biases. To ensure saturated carrier drift, the electric field must exceed 1 × 10^6^ V/m, while remaining below 1 × 10^7^ V/m to prevent ionization in Ge [30]. Accordingly, the reverse bias voltage of the P-Ge electrode is set to 1 V, while the P-Si electrode is set to 0 V. Figure 3e depicts the electric field along the black dashed line in N-Si at a 6.6 V bias voltage, showing a field greater than 3 × 10^7^ V/m (avalanche field threshold of silicon) and effectively confining the avalanche region within the Si layer [31].

Through optical structure optimization, the device enables efficient integration with Si_3_N_4_–based WDM components. Concurrently, electric field optimization allows independent control, enabling avalanche multiplication in Si while achieving optical absorption in Ge. This design fully exploits the high absorption of Ge in the O-band and leverages the low impact ionization coefficient ratio of Si to effectively suppress device noise. Furthermore, by reducing the carrier transit distance, the APD OE bandwidth is further enhanced. Taken together, these optimizations result in a device exhibiting excellent optoelectronic performance.

## 3. Simulation Results

**Figure 4:**
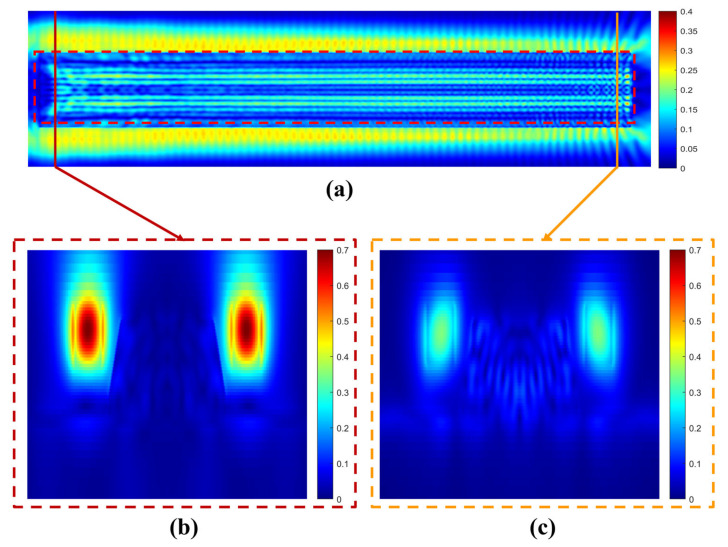
(**a**) Top view of the Si-Ge APD. (**b**) Cross-sectional optical field at the input of the Si_3_N_4_ waveguide. (**c**) Cross-sectional optical field at the output of the Si_3_N_4_ waveguide.

First, the optical field distribution of the APD at a wavelength of 1310 nm was simulated using FDTD. Figure 4a shows the top view of the optical field distribution, with the Ge region indicated by the red dashed box. In this APD coupling design, light is coupled from both sides, producing a uniform electric field distribution in the Ge region and thereby enhancing photodetection efficiency. Figure 4b,c present the cross-sectional optical field distributions at the input and output positions, respectively. The results indicate that the majority of the light in the Si_3_N_4_ waveguide is absorbed by Ge, with only a small fraction leaking outside the Ge region.

**Figure 5:**
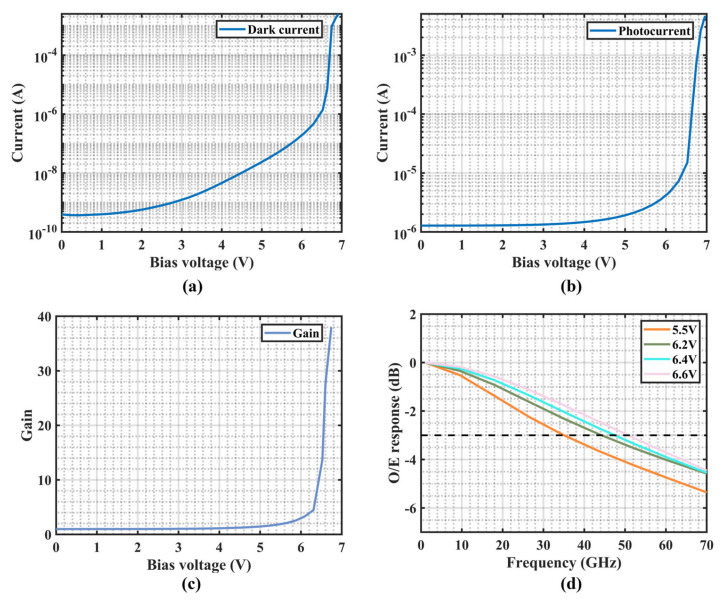
Performance of the Si-Ge APD. (**a**) Dark current as a function of bias voltage. (**b**) Photocurrent as a function of bias voltage. (**c**) Gain as a function of bias voltage. (**d**) Bandwidth as a function of bias voltage.

Based on the theoretical analysis described above and following the optical field analysis, the reverse bias voltage of the P–Ge electrode was set to 1V, while the P–Si electrode was set to 0 V. Under this condition, further simulations were carried out to obtain the dark current, photocurrent, gain, and small-signal response of the APD. Figure 5a shows the dark current as a function of bias voltage. The dark current of the Si-Ge APD initially increases slowly with rising reverse bias and accelerates as the breakdown voltage is approached. The device undergoes breakdown at 6.8 V, where the dark current exceeds 100 μA—significantly lower than the ~25 V breakdown voltage of conventional separate absorption charge multiplication-structured APDs [32]. Here, the breakdown voltage of Ge/Si APD is defined as the voltage at which the dark current reaches 100 μA [33]. At an operating voltage of 6.6 V, the dark current of the device is 7.31 μA. Figure 5b presents the photocurrent as a function of bias voltage under an input optical power of –21 dBm. At a reverse bias of 2 V, the device operates at unity gain, corresponding to a responsivity of 0.85 A/W. It can be observed that the photocurrent increases progressively with bias voltage, and the growth rate accelerates markedly once the avalanche threshold is exceeded. Figure 5c presents the gain curve, calculated according to Equation (1), with a maximum gain of 27 at 6.6 V reverse bias voltage. The avalanche multiplication gain *G*(*V*) is defined as the difference between the photocurrent (*I_p_*) and the dark current (*I_d_*) at a given bias voltage, normalized to the corresponding value at the unity gain point *V*_0_:

G(*V*) = (*I_p_*(*V*) − *I_d_*(*V*))/(*I_p_*(*V*_0_) − *I_d_*(*V*_0_)), (1)

Figure 5d presents the small-signal simulation results, showing that the 3-dB OE bandwidth increases with reverse bias, reaching 51 GHz at 6.6 V. Under the same bias condition, the gain of the device is 27. Thus, the GBP, obtained as the product of gain and bandwidth, is 1377 GHz, confirming the excellent performance of the proposed APD and its suitability for high-speed data transmission.

**Figure 6:**
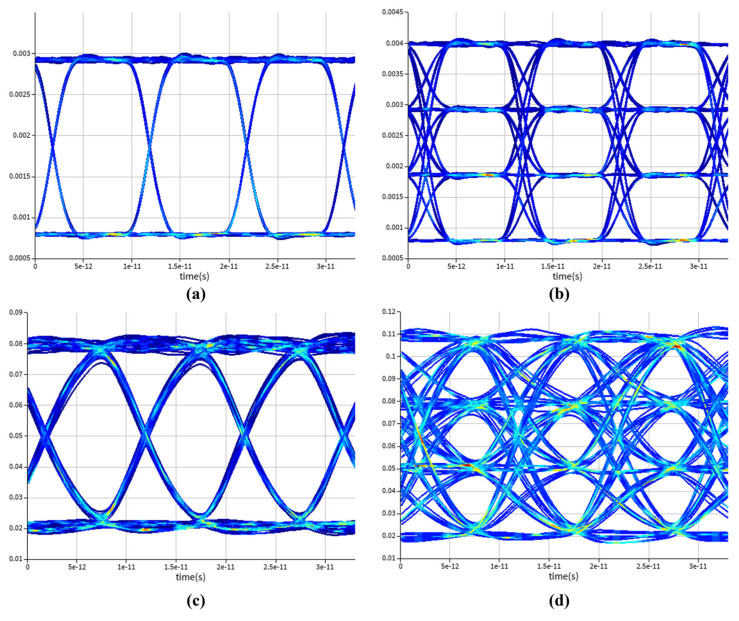
For the Si-Ge APD, eye diagrams for 100 Gbps NRZ signals. (**a**) Original optical signal eye diagram. (**c**) Eye diagram after detection, eye diagrams for 200 Gbps PAM4 signals. (**b**) Original optical signal eye diagram. (**d**) Eye diagram after detection.

Finally, to verify the signal transmission integrity, a system-level eye diagram simulation was performed, and the results are shown in Figure 6. At a bias voltage of 6.6 V and a temperature of 25 °C, the device exhibits a dark current of 7.31 μA, a gain of 27, and a 3-dB bandwidth of 51 GHz. Under these conditions, the static power consumption of the device is 48.2 μW, while the dynamic power consumption reaches 1.2 mW at an input optical power of −21 dBm. The APD under these operating conditions was used for the system-level simulation. A 1310 nm laser source was employed as the optical input, and a high-speed radio frequency (RF) signal was applied through an intensity modulator. The modulated optical signal was then injected into the APD to generate eye diagrams. To fully demonstrate the high-speed transmission capability of the proposed APD, no additional noise source was included in the simulation. Figure 6a,c show the input optical eye diagrams for 100 Gbps NRZ and 200 Gbps PAM4 signals, respectively, while Figure 6b,d present the corresponding output eye diagrams after the APD detection, with signal-to-noise ratios (SNRs) of 12.5 dB and 3.49 dB, respectively. The results indicate that the high-speed modulated signals maintain clear eye openings after passing through the APD, demonstrating excellent signal integrity during high-speed transmission. Compared with the Si-Ge PD without avalanche gain, the internal multiplication effect of the APD contributes to an improved SNR under low-input optical power.

## 4. Discussion

In recent years, various advanced modulation formats and multidimensional multiplexing techniques have been widely adopted to further enhance the transmission rate and capacity of optical communication systems, fully exploiting their bandwidth potential. Currently, Si_3_N_4_ waveguides can be efficiently integrated on SiPh platforms, enabling direct coupling of optical signals into devices. However, most existing APDs still rely on Si waveguide inputs, necessitating a converter between the Si_3_N_4_ and Si waveguides [16], which not only increases system loss but also exacerbates polarization dependence. Therefore, direct coupling via Si_3_N_4_ waveguides remains a promising avenue for further exploration.

Meanwhile, in high-speed optical interconnect systems such as data centers, the bias voltage of APDs remains a critical bottleneck. As summarized in Table 1, conventional APDs typically require operating voltages of 10 V or higher [2,17,34,35], which severely limits further integration. To address this, considerable efforts have been made to reduce the operating voltage. For instance, a three-terminal APD proposed in 2019 lowered the operating voltage to 6 V; however, due to its doping configuration, the device’s capacitance and other electrical characteristics were affected, further limiting the bandwidth. Additionally, its responsivity was relatively low, with only 0.48 A/W at a reverse bias of 2 V in the C-band [26].

Compared with previous three-terminal designs, the optimizations in this work focus on two key aspects: (1) employing lateral coupling through a Si_3_N_4_ waveguide, which mitigates optical absorption induced by heavy doping, thereby preventing responsivity degradation while also simplifying integration with Si_3_N_4_ waveguide-based wavelength-division multiplexers (WDMs); and (2) introducing doping beneath the Ge absorption region to shorten the carrier collection path, which contributes to bandwidth enhancement.

Although Si_3_N_4_ waveguide integration on Si platforms is already mature and the optical structures are relatively easy to fabricate, our design introduces an innovative P^+^ doping layer in the Si waveguide beneath the Ge region, along with an additional electrode. Compared with conventional two-electrode APDs [36,37], this device presents certain fabrication challenges; thus, the electrode placement still has room for optimization, and further refinement of the doping profile to simplify fabrication constitutes future work. Despite these challenges, the proposed APD remains highly attractive for achieving lower operating voltages.

## 5. Conclusions

In this study, we simulated the design of an O-band three-terminal Si-Ge APD, where the optical signal is laterally coupled into the Ge absorption region via a Si_3_N_4_ waveguide. At a reverse bias voltage of 2 V, the device exhibits a responsivity of 0.85 A/W; under 6.6 V bias, the bandwidth reaches 51 GHz with a DC gain of 27, yielding a GBP of 1377 GHz. Eye-diagram simulations for 100 Gbps NRZ and 200 Gbps PAM4 signals further confirm its high-speed performance. The Si-Ge APD is highly compatible with Si_3_N_4_ WDM and can achieve avalanche multiplication at relatively low bias voltage, demonstrating broad potential for high-speed optical communication applications.

## Figures and Tables

**Table 1:** Summary of the state-of-the-art Si-Ge APDs.

Reference	Avalanche Region	λ (nm)	V_bias_ (V)	Id (μA)	Gain	BW (GHz)	GBP (GHz)	Bit Rate (Gbps)
[2]	Si	1310	−12.5	~8.4	244	29	7078	112 (PAM4)
[17]	SI	1310	−12	~100	11	27	300	50 (OOK)
[26]	Si	1550	−6	~10	15	18.9	284	35 (OOK)
[34]	Si	1310	−17	~0.12	-	56	-	-
[35]	Si	1310	−11	~3.5	8	17	136	56 (OOK)
This Work *	Si	1310	−6.6	7.31	27	51	1377	200 (PAM4)

* Simulation results.

## Data Availability

Data underlying the results presented in this paper are not publicly available at this time but may be obtained from the authors upon reasonable request.
